# Inborn errors of immunity: a structured model for paediatric-to-adult transition of care

**DOI:** 10.3389/fimmu.2026.1813944

**Published:** 2026-05-11

**Authors:** Adele Civino, Federico Diomeda, Concetta D’Orio, Stefania Antonacci, Giulia Loiacono, Angelo Vacca, Baldassarre Martire

**Affiliations:** 1 Pediatric Rheumatology and Immunology Unit, ‘Vito Fazzi’ Hospital, Lecce, Italy; 2 Dietetics and Clinical Nutrition Unit, ‘Vito Fazzi’ Hospital, Lecce, Italy; 3 Pharmaceutical Department, ASL Bari, Bari, Italy; 4 Unit of Internal Medicine “Guido Baccelli”, Department of Precision and Regenerative Medicine and Ionian Area (DiMePRe-J), University of Bari Aldo Moro, AUOC Policlinico Di Bari, University Hospital Policlinico Di Bari, Bari, Italy; 5 Pediatrics and Neonatology Unit, Maternal-Infant Department, Monsignor A. R. Dimiccoli Hospital, Barletta, Italy

**Keywords:** immunoglobulin replacement therapy, inborn errors of immunity, paediatric-to-adult healthcare transition, primary immunodeficiency, transition of care

## Abstract

The transition of care from paediatric to adult healthcare services is a vulnerable period for patients with inborn errors of immunity (IEI), a heterogeneous group of primary immunodeficiency disorders characterized by chronic immune dysfunction and significant morbidity. With advances in diagnosis and treatment, an increasing number of patients with IEI are now reaching adulthood, making the management of this transition a growing clinical priority. Inadequate transition is associated with medical complications, treatment non-adherence, discontinuity of care, increased healthcare utilization, and poorer long-term outcomes. Despite growing awareness, condition-specific and standardized transition guidelines for IEI remain largely absent, leaving clinicians without a clear framework to guide this critical phase. In this context, the present review proposes a structured, multidisciplinary transition protocol designed specifically for paediatric patients with IEI to support therapeutic adherence, continuity of care, and patient well-being. This practice-informed narrative review integrates a non-systematic appraisal of the available literature, expert clinical consensus, and the practical experience of a dedicated Italian centre. Central to this model are the early identification of patients eligible for transition, beginning around age 14, and the gradual involvement of a multidisciplinary team, with shared responsibility between paediatric and adult specialists throughout an overlap period of at least three years. Clear documentation, including a standardized clinical dossier and final transition report, along with measurable process and outcome indicators such as infection rates, follow-up adherence, and patient-reported quality of life, ensures continuity of care and ongoing support. The framework also addresses immunoglobulin replacement therapy options, including intravenous, subcutaneous, and facilitated subcutaneous immunoglobulin, which each offer distinct advantages depending on patient needs and life stage. Patient and caregiver empowerment, timely management of comorbidities, and regular reassessment of individual needs are core components. Although resource-intensive, the proposed model is designed to be adaptable to different healthcare settings. Limitations, including reliance on multidisciplinary resources and the absence of prospective validation, are acknowledged, and future research to evaluate its impact on clinical outcomes is warranted. Through coordinated, standardized strategies, this work aims to bridge the gap between paediatric and adult healthcare, reducing care discontinuity and supporting successful long-term disease management in adulthood.

## Introduction

Inborn errors of immunity (IEI) encompass a broad and heterogeneous spectrum of rare genetic conditions characterized by chronic dysfunction of the immune system. IEI are caused by genetic defects affecting innate and adaptive immunity, non-hematopoietic cell-mediated immunity, or immune regulation (1). Patients with IEI are predisposed to recurrent infections, immune dysregulation, allergy, granulomatosis, bone marrow failure, and malignancy (2). Although each disorder is rare, collectively they represent a growing global public health challenge (1).

Since 1970, an international expert committee of clinicians and scientists, initially under the World Health Organization and now under the International Union of Immunological Societies (IUIS), has continuously updated the classification of IEIs. Advances in diagnostic tools, particularly next-generation sequencing, have led to a marked increase in the number of identified cases and genetic defects over the past two decades (3). In 2024, the IUIS Expert Committee reported 63 new IEIs, bringing the total to 555 (2). The IUIS classification organizes IEI into ten major groups with overlapping phenotypes: combined immunodeficiencies (CIDs), combined immunodeficiencies with syndromic features, predominantly antibody deficiencies (PADs), diseases of immune dysregulation, congenital defects of phagocytes, defects in intrinsic and innate immunity, autoinflammatory diseases, complement deficiencies, bone marrow failure, and phenocopies of IEI (2, 4).

A recent study estimates the prevalence of IEI at 6 per 10, 000 people in the United States, or approximately 150, 000–200, 000 patients (5). According to the 2019 IUIS classification, data from the Italian Primary Immunodeficiency Network (IPINet) show that the minimal prevalence of primary immunodeficiencies in Italy is 5.1 per 100, 000 people, with a mortality rate of 4.2%, consistent with other European registries (6).

Many patients with IEI experience a prolonged diagnostic and treatment odyssey before receiving an accurate diagnosis, leading to distress, delayed care, and reduced quality of life (7). This course often implies difficult interactions with the healthcare system, leaving psychological and emotional scars that may undermine trust in healthcare professionals. Such mistrust becomes particularly apparent during the transition from paediatric to adult care, a phase already associated with heightened anxiety and uncertainty (7).

The clinical spectrum of IEI is highly heterogeneous, and the management of young adults with these chronic conditions, once often fatal in childhood, has become an emerging challenge. Advances in diagnosis and treatment have increased the number of patients reaching adulthood (8). However, specific guidelines on transition of care remain limited, despite their critical role in ensuring continuity and quality of long-term follow-up (8). Studies also highlight psychosocial challenges, such as anxiety over leaving paediatric care, uncertainty about adult healthcare systems, and social isolation (7). Transition planning is complex, requiring collaboration among patients, families, and multiple specialists (9). Establishing a dedicated multidisciplinary team that bridges paediatric and adult services is crucial to ensure continuity of care, effective communication, and a supportive transition experience (7–9).

Given the growing number of patients with IEI reaching adulthood and the need to ensure continuity and quality of long-term follow-up, this review aims to define a standardized transition-of-care model for these patients. This article presents a practice-informed narrative review, integrating clinical experience, available literature, and expert consensus, and proposes a single-centre transition care model for patients with IEI. It does not constitute a systematic review and was not conducted according to PRISMA guidelines.

## Methods

This article is a narrative review informed by the clinical experience of the authors, who are practising specialists in paediatric and adult clinical immunology. Rather than following a systematic or pre-registered literature search protocol, the review draws on a non-systematic, selective appraisal of relevant English-language publications, with no formal screening or PRISMA-compliant selection process applied. Key references were identified through PubMed and Google Scholar using the search terms “inborn errors of immunity, ” “primary immunodeficiency, ” “transition of care, ” and “long-term follow-up, ” supplemented by expert knowledge, relevant international society documents (IUIS, IPINet), and cross-referencing of bibliographies. The selection of literature was guided by clinical relevance and expert judgement rather than predefined eligibility criteria; accordingly, no formal data extraction or quality assessment of individual studies was performed. The authors synthesized the available evidence through expert discussion and critical appraisal, with the aim of identifying key challenges, existing gaps, and current best practices in the transition from paediatric to adult care for patients with IEI. The diagnostic, therapeutic, and care pathway developed at the Vito Fazzi Hospital in Lecce, Italy, for paediatric and adult patients with congenital immune disorders is presented as an illustrative example of a practical, single-centre model for managing transitional care (10).

## Results

### Implementation and effectiveness of the transition model

Transition of care is defined as the purposeful, planned movement of adolescents and young adults with chronic conditions from child- and family-centred to adult-oriented healthcare systems (11). This pathway is particularly complex for youths with special healthcare needs, such as those with IEI, due to the involvement of multiple specialists and lifelong disease management requirements (12). As these patients move from paediatric to adult care, challenges commonly arise in maintaining continuity of care, managing new healthcare relationships, and developing self-management skills (13). The transition period is further complicated by concurrent developmental, social, and psychological changes, which can increase vulnerability to poor adherence, loss to follow-up, and higher morbidity and mortality (7).

Despite increasing attention to healthcare transition in chronic diseases, research specifically addressing IEIs remains limited. A recent European survey showed that transition services for children with IEI are available in many countries, but standardized guidelines to support best practices are still lacking (14).

IPINet recently published disease-specific guidelines for the transition of care in patients with IEI, based on clinical experience and expert consensus. The guidelines highlight that a structured transition of care process is essential to ensure proper long-term follow-up and optimal management of both persistent and emerging clinical issues (8). The transition process should be based on shared, patient- and family-centred decision-making within a coordinated, multidisciplinary team. Psychological support is considered fundamental to help patients manage the emotional and psychosocial challenges of chronic disease, particularly during the transition period. For adolescents with undiagnosed conditions, diagnostic re-evaluation is recommended before leaving paediatric care. The transition process should ideally begin around age 14 and conclude by age 25, including at least a three-year overlap period during which paediatric and adult specialists jointly follow the patient to ensure continuity of care (8).

Accordingly, this review proposes a transition protocol aimed at establishing a multidisciplinary model for managing patients as they move from childhood to adulthood, a framework intended to serve as a reference for the institution and to be adaptable to other disease pathways.

The pathway is structured around a series of general and specific objectives, as follows:

- Early identification of patients eligible for transition and the provision of gradual, structured support through the progressive involvement of the transition team.
- Ensuring continuity and coordination of care, including therapeutic reconciliation, to maintain an integrated and multidisciplinary clinical pathway across the paediatric–adult interface.
- Promoting collaboration among transition specialists through structured opportunities for discussion and shared decision-making, aimed at prevention and timely referral to appropriate care settings.
- Empowering both patients and healthcare professionals to enhance treatment adherence and self-management skills.
- Improving access to services through clear information tools, such as a formal service charter and dedicated transition pathway.
- Identifying and monitoring specific process and outcome indicators closely linked to the transition phase.

### Transition of care between paediatric and adult specialists

Once contact has been established between the paediatric immunologist and the adult specialist, the following steps are recommended:

- Scheduling the transition visit through a dedicated calendar.
- Conducting an initial joint visit with the adult specialist, preceded as needed by the exchange of documentation or an in-person meeting to define the transition care plan.

If a joint visit is not feasible due to emergencies or logistical constraints, the transition should proceed through the exchange of clinical documentation.

During the first joint meeting, it is recommended to:

- Introduce both specialists to the patient and caregivers.
- Share all relevant clinical information.
- Assess psychosocial needs and care requirements.

Following the initial meeting, the transition should continue under the shared responsibility of both paediatric and adult specialists. Periodic check-ups, along with any necessary diagnostic or instrumental evaluations, should be carried out in accordance with a jointly defined care plan. Throughout this process, any issues that emerge during follow-up should be promptly identified and reassessed collaboratively, with additional specialists involved when required for the management of comorbidities.

Frailty and complex cases require particular attention during transition. Any frailty identified should be actively addressed, and in situations characterized by significant clinical or social complexity, a multidisciplinary approach is recommended. Depending on the specific needs of the patient, local healthcare services may be engaged through chronic disease management programs, and when appropriate, social services should be involved to provide support for frailty or disability.

### Transition reporting and standardized clinical documentation

At the end of the transition period, it is recommended that a comprehensive report be prepared and shared among the specialists involved, and that a satisfaction questionnaire be administered to the young patient.

To ensure optimal management of the patient’s condition during and after transition, it is recommended to establish a shared language among all specialists involved. For this purpose, a standardized document containing a core set of minimum information (clinical, care-related, social, and psychological) should be prepared and maintained. Given the complexity and heterogeneity of the information to be transferred, it is recommended that transition documentation be complete, consistent, and uniform.

Information sharing between paediatric and adult specialists is recommended to take the form of a clinical dossier, the final transition report. This report should summarize the clinical pathway followed and provide a documentary account of the contents of the clinical dossier.

In the paediatric phase, the report should include:

- Personal and family medical history, including genetic or familial predispositions and associated comorbidities.
- The onset and course of the disease, along with diagnostic and therapeutic milestones.
- The role of parents and the patient’s progressive development of autonomy and awareness of clinical condition.
- An assessment of the patient’s readiness to manage a chronic condition independently or with caregiver support.

During the transition to adulthood, the report should include:

- A clinical assessment based on the patient’s history and current status.
- Planning for future follow-up and related treatment recommendations.

The transition process summary is presented in **Table 1**.

**Table 1:** Transition process summary.

No.	Section	Details
1	Individualized, Stepwise, and Gradual Approach	The transition process should be tailored to each patient’s psychological maturity and readiness. The transition period usually begins between ages 14 and 16 but may be adjusted according to the patient’s individual needs.
2	Key Personnel in the Transition	Transition Coordinator: paediatric immunologist responsible for supervising patient care and coordinating the transition. Transition Case Manager: Specialized nurse who meets patients entering the transition phase and monitors their progress.
3	Transition Team	A multidisciplinary team of specialists involved in the patient’s care. The team holds at least three key meetings: First meeting (ages 14–16): Case discussion between paediatric and adult specialists. • Introduction of specialists to the patient and caregivers. • Sharing of clinical information. • Assessment of specific psychosocial and care needs. Second meeting: Intermediate shared review of progress. Third meeting: Completion of the transition process, followed by a joint report and satisfaction questionnaire.
4	Core Elements of the Transition	Establish a formal transition policy. Implement monitoring and tracking systems. Assess transition readiness. Plan the transition process. Initiate and complete the transfer of care.
5	Family Integration	Provide support for parents as needed. Offer reproductive health education and genetic counselling. Support siblings who may experience emotional challenges. Consider the family burden associated with frequent medical appointments.
6	Implementation Strategies	Adopt a sequential rather than simultaneous transition to multiple adult physicians. Schedule regular briefings between paediatric and adult care services. Organize nurse-led health education meetings involving young adults who have completed their transition.

### Pathway indicators for monitoring the transition process

It is recommended that specific pathway indicators be systematically employed to evaluate both the clinical effectiveness and overall quality of the transition process. Clinical effectiveness should be assessed by tracking the annual number of severe infections and the rate of hospital admissions, providing an objective measure of patient outcomes. Adherence to follow-up should be monitored by measuring the proportion of patients attending scheduled visits across the paediatric, transition, and adult phases, reflecting continuity of care. Patient-reported outcomes, such as quality of life, should be evaluated using validated instruments like the Pediatric Quality of Life (PedsQL) (31) or Common Variable Immunodeficiency Quality of Life (CVID_QoL) (30) questionnaires, tailored appropriately for children, adolescents in transition, and adults.

Together, these indicators create a comprehensive framework that not only facilitates the monitoring of clinical outcomes but also ensures continuity of care and supports the overall well-being of patients throughout the transition pathway.

### Immunoglobulin replacement therapies and transition of care in inborn errors of immunity

Established therapies for IEI have long relied on immunoglobulin replacement, antimicrobial prophylaxis, and hematopoietic stem cell transplantation (HSCT). However, advances in molecular genetics and immunopathology have led to the development of targeted and curative approaches, including gene therapy, cytokine modulation, small-molecule inhibitors, and monoclonal antibodies directed at specific immune pathways (15).

Antibody deficiency disorders are the most frequently diagnosed form of IEI, accounting for roughly half of all cases worldwide (16). In Italy, IPINet data show an even higher prevalence, with antibody deficiencies comprising 63% of diagnoses, followed by combined immunodeficiencies with syndromic features (22.5%) (6).

The primary therapeutic goal in antibody deficiency disorders is to prevent or reduce infectious episodes and manage autoimmune complications. Immunoglobulin (Ig) replacement therapy remains the cornerstone of treatment, providing passive antibodies that effectively protect against infections (17). Immunoglobulins can be administered either intravenously (IVIg) or via the subcutaneous route (SCIg) (14). While IVIg and SCIg are considered equally effective, SCIg is associated with fewer systemic adverse events, improved quality of life, and greater patient autonomy (18) (**Table 2**). Compared with IVIg, SCIg does not require venous access, may be less time-consuming, and can be self-administered at home or in a healthcare setting (19). The lower rate of systemic infusion-related reactions is largely attributable to dividing the total monthly dose into smaller, more frequent administrations (20). Although the unit acquisition cost is higher for SCIg than for IVIg, SCIg has been shown to be more cost-effective in the long term, primarily due to reduced indirect healthcare costs resulting from the avoidance of regular hospital day-care visits, infusion facilities, and nursing supervision (19).

**Table 2:** Comparison of IVIg, SCIg, and fSCIg.

Characteristic	IVIg	SCIg	fSCIg
Dosage	400–600 mg/kg every 3–4 weeks	100–200 mg/kg/week	400–600 mg/kg every 3–4 weeks
Volume per site	Up to 2 L	20–30 ml	Up to 600 ml
Administration route	Intravenous	Subcutaneous	Subcutaneous
Infusion time	>2 hours	1–3 hours (infusion pump) 5–15 min prefilled syringe	1–3 hours (infusion pump)
Setting	Hospital	Home	Home
Peak–trough variation	Greater variation	Reduced variation yields nearly constant Ig levels	Reduced variation yields nearly constant Ig levels
Adverse effects	Systemic (30%–40%)	Local (mild, 5%–15%)	Local (mild, 5%–15%)

IVIg, intravenous immunoglobulin; SCIg, subcutaneous immunoglobulin; fSCIg, facilitated SCIg (incorporating recombinant human hyaluronidase, rHuPH20).

Facilitated SCIg (fSCIg) employs recombinant human hyaluronidase to enhance subcutaneous tissue permeability, enabling larger infusion volumes and longer dosing intervals comparable to IVIg (19) (**Table 2**). These characteristics make facilitated immunoglobulin therapy particularly well-suited for young adults in transition, as it combines the flexibility and independence of subcutaneous administration with the dosing convenience of monthly intravenous therapy.

SCIg is generally well tolerated during the transition phase, and most patients who switch from IVIg to SCIg choose to continue SCIg as their long-term therapy (21). For patients transitioning to new immunoglobulin replacement therapy formulations, treatment should be individualized, allowing flexibility in infusion parameters to accommodate patient needs (22). Transition from IVIg to SCIg, or initiation of SCIg in treatment-naïve patients, should occur initially under supervision in a specialized facility or by an experienced home infusion nurse (20). Before self-administration at home, patients must demonstrate proper technique and tolerability of SCIg or fSCIg therapy under medical supervision (20).

### Monitoring tool

To address the risk of fragmented care during the transition process, we propose a structured monitoring tool (**Table 3**) summarising key clinical, laboratory, and psychosocial parameters across different transition stages. Similar structured approaches have been successfully implemented in other chronic paediatric conditions, but remain lacking in IEI. This flow sheet is intended to support continuity of care, improve adherence, and facilitate communication between paediatric and adult healthcare providers.

**Table 3:** Structured transition monitoring flow sheet for IEI patients.

Domain	Early adolescence (12–14 yrs)	Mid adolescence (14–16 yrs)	Late adolescence (16–18 yrs)	Post-transition (Adult care)
Clinical Assessment	Disease history review; infection frequency; autoimmune manifestations	Update clinical status; identify emerging complications	Comprehensive clinical summary for transfer	Annual review; reassess disease activity, comorbidities, and complications
Laboratory Monitoring	Baseline immunological profile; Ig levels; lymphocyte subsets	Repeat key labs; monitor trends	Pre-transfer lab summary	Ig trough levels every 3–6 months; CBC, liver and renal function annually; lymphocyte subsets as clinically indicated
Organ-Specific Evaluation	Baseline lung, GI, liver assessment (if applicable)	Targeted reassessment based on disease	Pre-transfer organ status documentation	Annual pulmonary function tests; imaging if bronchiectasis; GI and hepatic follow-up as indicated
Immunoglobulin Replacement Therapy	Confirm IgRT regimen (IVIg/SCIg/fSCIg); educate patient and caregiver on administration	Reassess route (consider switch to SCIg/fSCIg for home use); verify dose and tolerability	Optimise IgRT for adult lifestyle; document current product, dose, and route for adult team	Maintain target trough levels; reassess route and setting (home vs. clinic) at each visit; adjust dose with weight/clinical change
Therapy Adherence	Caregiver-led education; introduce importance of adherence	Progressive patient involvement; adherence check at each visit	Patient autonomy in treatment management	Monitor missed infusions; use validated adherence tools; address barriers (work, travel, access)
Infection/Complication Surveillance	Education on warning signs	Monitor for disease-specific complications	Risk stratification before transfer	Record annual infection rate; screen for autoimmune, granulomatous, and malignant complications; prompt specialist referral
Vaccination Status	Review vaccination history; plan updates	Evaluate response to vaccines (if applicable)	Pre-transfer vaccination review	Adult vaccination schedule; annual influenza; pneumococcal and meningococcal boosters per guidelines
Psychosocial Aspects	Family-centred care; introduce transition concept	Assess psychological readiness; school/social impact	Promote autonomy; address transition anxiety	Screen for anxiety/depression (validated tools); support work/university integration; involve mental health services if needed
Transition Readiness	Initial readiness assessment	Structured transition planning	Final transfer plan; identify adult provider	Evaluate successful transfer; confirm patient is engaged with adult service within 3 months
Documentation/Data Sharing	Start structured medical summary	Update shared documentation	Complete transition report (“medical passport”)	Maintain accessible longitudinal record; update at each visit; share with patient and all providers

IEI, inborn errors of immunity; IgRT, immunoglobulin replacement therapy; IVIg, intravenous immunoglobulin; SCIg, subcutaneous immunoglobulin; fSCIg, facilitated subcutaneous immunoglobulin; CBC, complete blood count; GI, gastrointestinal.

## Discussion

Available evidence suggests that insufficient transition of care interventions are associated with higher rates of medical complications, reduced health and well-being, treatment non-adherence, discontinuity of care, patient dissatisfaction, increased emergency and hospital utilization, and greater healthcare costs (9). Although many countries offer transition services for children with IEI, clear and standardized, condition-specific guidelines remain largely absent (9, 14, 23).

Therapeutic adherence is a central challenge during the transition phase for individuals with IEI. A recent study showed that medical non-adherence, reported in up to 45% of cases, significantly contributes to adverse outcomes (24). Immunoglobulin replacement therapy remains the cornerstone of management; however, because its effects are temporary and require regular infusions, sustained adherence is crucial (25). Transition is also a period in which treatment modalities may evolve. Paediatric patients often begin with IVIg, especially when parents are uncomfortable performing frequent subcutaneous infusions (25). As patients mature, switching to home-based SCIg often better matches their lifestyle, offering flexibility, greater autonomy, and compatibility with academic or work schedules (25). Consequently, periodic reassessment of both the administration route and care setting is essential to support long-term adherence (25). A recent study demonstrated that fSCIg is a feasible, safe, and highly adaptable option for children and adolescents up to 18 years of age with primary or secondary immunodeficiencies, supporting its use every 3–4 weeks in both home and hospital settings (26). The study reported flexible infusion parameters, successful transition to home administration within 6 months, high adherence to scheduled dosing, and consistent delivery of full planned doses without technical issues or premedication (26).

Transition is further complicated by the heterogeneity of IEI and associated comorbidities such as autoimmunity, allergy, chronic lung disease, malignancy, and late effects of hematopoietic cell transplantation or gene therapy (24). Many adult subspecialists may be unfamiliar with IEIs or their organ-specific complications, underscoring the importance of consensus transition of care guidelines that help primary care clinicians and adult subspecialists anticipate complications and late effects. Multidisciplinary visits involving relevant specialties can be critical for coordinated and comprehensive care (24). Patients with chronic illnesses typically experience an average gap of 20 months from their last paediatric-focused visit to their first adult-focused visit (27). However, a recent single-center study showed that a planned multidisciplinary approach significantly minimized interruptions during this vulnerable period, enabling patients to establish adult immunology care in an average of just 3 months (24). Thus, based on clinical experience, early identification of patients eligible for transition, combined with gradual support from the transition team, may help support continuity of care and timely interventions. The process is maintained under shared responsibility, with periodic check-ups, necessary evaluations, and collaborative management of emerging issues. This approach aligns with recent recommendations suggesting that young patients undergo a period of joint follow-up by both paediatric and adult clinics in the year preceding transfer (23).

Insufficient support during transition can lead to increased urgent care and emergency department use, fragmented care, higher costs, and poorer outcomes, particularly when new practitioners are unfamiliar with the patient’s complex IEI history (28). The integration of standardized transition documentation, including a clinical dossier and final transition report, ensures accurate communication of the patient’s medical, psychosocial, and treatment history. This approach supports optimal long-term management, enables empowerment of patients and caregivers, and allows early detection and management of frailty or other complex clinical and social needs.

Previous research has identified several indicators and outcomes of successful transition, focusing on quality measures such as satisfaction, transition education and planning, continuity of care, and self-management or self-efficacy (29). Building on this, the present narrative review proposes the use of specific pathway indicators to evaluate the transition process, including clinical effectiveness, adherence to follow-up, and patient-reported quality of life. Together, these measures provide a comprehensive framework to monitor outcomes, ensure continuity of care, and support patient well-being throughout the transition.

Several limitations of this work must be acknowledged. First, regarding the nature of the literature review: this article is a narrative review based on a non-systematic, selective appraisal of the literature, guided by expert judgement rather than pre-specified eligibility criteria. No systematic search strategy, formal screening process, or PRISMA-compliant methodology was applied. As a result, the review may be subject to selection bias, with a potential tendency to favour publications that support the proposed model. The possibility of publication bias — whereby studies with positive or confirmatory findings are more likely to have been retrieved and cited — cannot be excluded. Readers should therefore interpret the evidence synthesis presented here as expert-informed rather than exhaustive or systematic. Second, regarding the transition model itself: the protocol described is derived from the clinical experience of a single Italian tertiary centre. Its design is institution-specific and resource-intensive, relying on dedicated multidisciplinary teams, structured scheduling, and sustained follow-up capacity. These features may limit its direct applicability to healthcare settings with fewer specialist resources, different structures, or varying levels of institutional support. Third, the proposed model has not undergone prospective external validation. While process and outcome indicators are recommended, their actual impact on long-term clinical outcomes, patient satisfaction, treatment adherence, and care continuity has not yet been formally evaluated in independent cohorts. The pathway therefore requires prospective assessment and adaptation before it can be considered generalisable beyond its current institutional context. Future multicentre studies comparing structured transition models against standard care, including patient-reported outcomes and health economic analyses, would substantially strengthen the evidence base for this field.

## Conclusions

The model presented here highlights a structured approach to transition of care for paediatric patients with IEI, addressing a critical period often associated with medical complications, poor adherence, fragmented care, and increased healthcare utilization. Condition-specific guidelines for IEI remain limited, underscoring the need for tailored strategies. Given the complexity of IEI and associated comorbidities, the model emphasizes multidisciplinary coordination, early patient identification, shared responsibility between paediatric and adult specialists, and structured documentation to ensure continuity of care and timely interventions. Monitoring outcomes through clinical effectiveness, adherence, and quality of life indicators provides a framework to evaluate success and support long-term patient well-being.
